# Mind Gaps and Bone Snaps: Exploring the Connection Between Alzheimer’s Disease and Osteoporosis

**DOI:** 10.1007/s11914-023-00851-1

**Published:** 2024-01-18

**Authors:** Hannah S. Wang, Sonali J. Karnik, Tyler J. Margetts, Lilian I. Plotkin, Alexandru Movila, Jill C. Fehrenbacher, Melissa A. Kacena, Adrian L. Oblak

**Affiliations:** 1 Department of Orthopaedic Surgery, Indiana University School of Medicine, Indianapolis, IN 46202 USA; 2 Department of Anatomy, Cell Biology & Physiology, Indiana University School of Medicine, Indianapolis, IN 46202 USA; 3 Indiana Center for Musculoskeletal Health, Indiana University School of Medicine, Indianapolis, IN 46202 USA; 4 Richard L. Roudebush VA Medical Center, Indianapolis, IN 46202 USA; 5 Department of Biomedical Sciences and Comprehensive Care, Indiana University School of Dentistry, Indianapolis, IN 46202 USA; 6 Department of Pharmacology and Toxicology, Indiana University School of Medicine, Indianapolis, IN 46202 USA; 7 Stark Neurosciences Research Institute, Indiana University School of Medicine, Indianapolis, IN 46202 USA; 8 Department of Radiology & Imaging Sciences, Stark Neurosciences Research Institute, Indiana University School of Medicine, Indianapolis, IN 46202 USA

**Keywords:** Alzheimer’s disease, Osteoporosis, Wnt/β-catenin, Fracture healing, AI, Artificial intelligence, ChatGPT

## Abstract

**Purpose of Review:**

This comprehensive review discusses the complex relationship between Alzheimer’s disease (AD) and osteoporosis, two conditions that are prevalent in the aging population and result in adverse complications on quality of life. The purpose of this review is to succinctly elucidate the many commonalities between the two conditions, including shared pathways, inflammatory and oxidative mechanisms, and hormonal deficiencies.

**Recent Findings:**

AD and osteoporosis share many aspects of their respective disease-defining pathophysiology. These commonalities include amyloid beta deposition, the Wnt/β-catenin signaling pathway, and estrogen deficiency. The shared mechanisms and risk factors associated with AD and osteoporosis result in a large percentage of patients that develop both diseases. Previous literature has established that the progression of AD increases the risk of sustaining a fracture. Recent findings demonstrate that the reverse may also be true, suggesting that a fracture early in the life course can predispose one to developing AD due to the activation of these shared mechanisms. The discovery of these commonalities further guides the development of novel therapeutics in which both conditions are targeted.

**Summary:**

This detailed review delves into the commonalities between AD and osteoporosis to uncover the shared players that bring these two seemingly unrelated conditions together. The discussion throughout this review ultimately posits that the occurrence of fractures and the mechanism behind fracture healing can predispose one to developing AD later on in life, similar to how AD patients are at an increased risk of developing fractures. By focusing on the shared mechanisms between AD and osteoporosis, one can better understand the conditions individually and as a unit, thus informing therapeutic approaches and further research. This review article is part of a series of multiple manuscripts designed to determine the utility of using artificial intelligence for writing scientific reviews.

## Introduction

This is one of many articles evaluating the utility of using AI to write scientific review articles on musculoskeletal topics [1]. The first draft of this review was written entirely by humans. Refer to this edition’s Comment paper for more information [2]. Alzheimer’s disease and related dementias (AD/ADRD) and osteoporosis are two diseases that are prevalent in our aging population, and they unfortunately have a deleterious impact on quality of life [3]. Individuals living with AD typically experience a progressive loss of cognition, while those with osteoporosis are at increased risk of developing fractures. Patients diagnosed with both AD and osteoporosis may experience decreased cognitive agility, decreased mobility, and decreased ability to take care of themselves. Research has shown that these two disease processes are more intertwined than previously thought—in fact, they share many of the same molecular pathways and risk factors, such as old age, lifestyle, and fractures [4, 5]. While not the focus of this review, it is important to note that bone-brain cross-talk may be important in this process. Indeed, Yuan et al. recently reviewed the role of bone-derived modulators and AD progression. They describe that bone-derived cells and secreted proteins interact with multiple organ systems including the central nervous system, and such cross-talk between systems is important in the progression of AD [6]. In the current review, the commonalities between AD and osteoporosis will be elucidated, and a discussion of AD and fractures will seek to uncover whether each of the diseases affects the onset and progression of the other. Uncovering the complex relationship between these conditions could have important implications for improving prognosis and quality of life for those afflicted, which will be especially significant for our aging population.

## Background on Alzheimer’s Disease and Related Dementias (AD/ADRD)

AD is the most common cause of dementia in the elderly and affects 6.7 million people in the USA, equaling roughly 1 in 9 individuals over the age of 65 [7]. Alzheimer’s disease and other dementias cost the USA $345B every year, with an additional estimated $339.5B in unpaid care, such as that provided by family and friends in the home [7]. AD is a deadly disease: deaths resulting from AD complications have doubled since 2000, and the 10-year survival rate for 70-year-old AD patients is half that of those without AD. AD is a multifactorial disease with many associated risk factors including advanced age, sex, genetic markers (e.g., apolipoproteinE4 (ApoE4) allele), traumatic head injuries, and environmental factors. Patients with AD present with multiple impairments including declines in cognition and memory and behavioral changes.

AD/ADRD is characterized by extracellular amyloid plaque deposition and intracellular neurofibrillary tangles in the medial temporal lobe of the brain, as well as widespread cerebral atrophy [8]. These pathological abnormalities result in many neurological changes in AD, which can be divided into two categories: positive lesions and negative lesions. Positive lesions are characterized by accumulations of abnormal deposits in the brain, such as amyloid plaques, and neurofibrillary tangles and negative lesions involve neuronal and synaptic loss. Abnormal deposition of beta-sheets has a strong correlation with dementia; beta-sheets provide the composition of fibrils, which aggregate to form amyloid plaques [9]. The transmembrane amyloid precursor protein (APP) is cleaved by proteolytic enzymes, yielding several varieties of amyloid beta (Aβ) monomers, including large and insoluble amyloid fibrils [10–12]. APP has been identified as a cause of early-onset AD when mutated [13]. The amyloid hypothesis posits that the degradation of the Aβ plaques is decreased with advanced age, thus leading to the aggregation of amyloid plaques. These amyloid plaques accumulate in chains of 39–43 amino acid Aβ peptides [14] throughout the brain causing neurotoxicity and inhibiting neural function, which can lead to cognitive impairment [8, 9, 12, 15]. Neurofibrillary tangles are hyperphosphorylated tau proteins and consist of accumulations of paired helical filaments that are characteristic of intracellular changes in AD [16]. Normally, tau acts as a scaffolding protein in microtubules to enrich axonal connections. Tau can undergo many post-translational modifications, such as monomethylation, acetylation, phosphorylation, and ubiquitination [17]. When tau protein becomes hyperphosphorylated, it begins to aggregate and loses its specificity for microtubules, impairing axonal function and causing neurodegeneration. The observed cerebral atrophy of negative lesions in AD is due to the loss of neurons and synapses throughout the brain, which may be more pronounced in the hippocampus and amygdala [8].

Despite the increasing prevalence of the disease, there is no cure for AD. Although the few drugs that have been approved by the FDA for AD are useful in temporarily alleviating symptoms, there has been little success in slowing or halting the progression of AD [17]. In July 2023, a new FDA-approved drug, lecanemab, showed a modest slowing of AD progression and reduced Aβ; however, debate is ongoing over the benefits in light of the undesirable side effects such as infusion-related reactions [18•].

## Background on Osteoporosis

Osteoporosis is a prevalent skeletal condition associated with bone fragility due to low bone mass and compromised bone structure. It is estimated that there are over 10 million individuals living with osteoporosis in the USA [19]. The disruption of bone architecture seen in osteoporosis is a result of greater rates of bone loss than bone formation, which reduces bone strength and leads to an increased risk of fractures [20]. Bone remodeling is a continuous process of replacing older bone material with new bone material, helping to repair microfractures and preventing the onset of macrofractures. However, this process becomes impaired with aging-induced increases in bone resorption and reductions in bone formation. With this impaired balance, the architectural structure of bone becomes weakened due, in part, to a significantly reduced mass combined with deleterious changes in bone structure such as cortical thinning, leading to an increased incidence of fractures and subsequent decline in daily functioning.

Osteoporosis typically goes undiagnosed until a fracture occurs. A fracture of the hip or vertebrae without any severe trauma is diagnostic of the disease. Osteoporosis can also be diagnosed using a metric known as the *T*-score. This scoring involves measuring the patient’s bone mineral density (BMD) with a dual X-ray absorptiometry (DXA) scan and comparing this measurement to the mean BMD of young healthy people aged 20–29. BMD accounts for 70% of bone strength, with an additional 20% coming from bone quality, which is currently unmeasurable [20].

## Overview of Alzheimer’s and Osteoporosis

While AD and osteoporosis seemingly affect very different organ systems, there are many commonalities between them. In fact, multiple AD mouse models have been shown to express an osteoporotic phenotype [21••]. In the current section, we will look at the underlying risk factors and pathways shared by AD and osteoporosis. It has been observed that osteoporosis and bone fracture occur at roughly twice the rate in AD patients compared to non-AD patients of comparable age [22]. Indeed, a previous cross-sectional study reported that individuals with AD were more likely to have sustained a hip fracture during their lifetime, have concurrent osteoporosis, and have fallen, as compared to individuals with no diagnosis of AD [23]. The risk factors between AD and osteoporosis that are shared include advanced age of the patients, poor nutrition, poor gait, impaired metabolism due to underlying co-morbidities, and sex-based differences in physiology.

Aβ has been implicated in the damage of bone tissue, as it has been shown that Aβ directly interacts with bone cells to increase bone resorption by osteoclasts and inhibit differentiation of osteoblasts, thus compromising bone architecture. APP, a transmembrane protein mentioned earlier as a precursor to amyloid plaques in the brain, is also expressed in osteoblasts and osteoclasts, two cell types important for bone remodeling. When certain mutations in APP occur, osteoblast differentiation is suppressed, preventing new bone growth and laying the foundation for osteoporosis [13].

Many pathways have been identified as having commonalities in both AD and osteoporosis. Studies have shown that patients with osteoporosis have an increased risk of developing AD compared to those without osteoporosis [24]. Furthermore, osteoporosis typically precedes a diagnosis of AD. This could indicate a pathophysiological link, which is not yet well understood.

While many observational studies have established an association between AD and osteoporosis, a recent two-sample Mendelian randomization study found that there was no distinct causal genetic link between the two conditions [25]. These researchers isolated potentially pleiotropic single nucleotide polymorphisms and found that the removal of such genes did not confer the development of osteoporosis or AD directly. It is worth mentioning that in addition to genetic links, environmental and physiological causes can be determinants of diseases, especially diseases associated with aging. As mentioned earlier and discussed in detail in the sections to follow, AD and osteoporosis share similar pathways and pathogenesis, which continue to require further investigation.

## Shared Pathways Between Alzheimer’s Disease and Osteoporosis

The Wnt/β-catenin signal transduction pathway regulates many cellular processes in the body, including cell survival [26]. In the brain, this pathway works to increase neuronal survival, promote neurogenesis, and regulate synaptic plasticity [26]. The Wnt/β-catenin signaling pathway has been linked to AD, as its normal activation serves to inhibit Aβ production and tau phosphorylation (p-tau) in the brain. In aging brains, Wnt/β-catenin signaling is downregulated, and this suppression is even greater in AD brains [27]. Loss of function of the Wnt co-receptor LRP6 has been shown to downregulate the Wnt/β-catenin signaling pathway and is associated with an increased risk of developing AD [28, 29] while contributing to the synaptic dysfunction and Aβ accumulation seen in AD [30].

The Wnt/β-catenin signaling pathway is also a critical player in the facilitation of bone formation. The loss of Wnt/β-catenin signaling in osteocytes, specifically β-catenin gene deletion, causes an elevation of both the number and activity of osteoclasts, leading to substantial bone loss [31]. Furthermore, osteoclasts stimulate osteoblast differentiation through the secretion of Wnt ligands and chemoattractants to aid in skeletal remodeling [32]. Osteoblastic cells in turn impact osteoclastogenesis through the expression of RANKL and OPG, which work to differentiate osteoclasts [33, 34]. Thus, the interplay between bone regeneration and remodeling involves cytokine signaling, including Wnt/β-catenin, RANKL, and OPG which at least the former has also been implicated in AD as described in more detail below.

Studies using mouse models of AD, rat neurons in vitro cultures, and samples from human Alzheimer’s patients have identified deficits in the Wnt/β-catenin signaling pathway that accounts for both the Aβ and tau pathogenesis seen in AD, as well as the characteristic bone loss of osteoporosis. The Wnt/β-catenin signaling pathway has been shown to facilitate bone formation and promote synapse formation in the brain [22], and the disruption of this pathway has been implicated in both the onset of osteoporosis and AD. In relation to AD, the inhibition of the pathway allows for the unregulated production of p-tau and Aβ, leading to the accumulation and deposition of these proteins [22, 35]. Upon accumulation of p-tau and Aβ, inflammatory pathways are activated, further inhibiting the Wnt/β-catenin signaling pathway and contributing to a vicious cycle of p-tau and Aβ deposition. Disruptions in Wnt/β-catenin signaling are typically seen prior to the onset of AD [22, 36]. Dengler-Crish and Elefteriou in 2019 hypothesized that a disruption in the Wnt/β-catenin signaling pathway causes peripheral accumulation of Aβ initially and that the positive-feedback loop of further accumulation leads to deposition in the central nervous system, contributing to the pathogenesis of AD [22].

## Angiogenesis

Angiogenesis is the process of forming new blood vessels from existing vasculature [37]. Angiogenesis is vital for proper bone repair, as it is involved in the development of new bone tissue and bone remodeling [38–41]. Angiogenesis is also a highly relevant process in AD, as the accumulation of amyloid plaques damages the cerebrovasculature.

A study using a transgenic mouse model (Tg APPsw) of amyloidosis found that the overexpression of APP may oppose angiogenesis, leading to decreased functional vasculature in the brain [14]. The impaired angiogenesis seen in AD patients leads to decreased capillary diameter, thinning of the capillary basement membrane, and atrophy of the cerebrovascular smooth muscle [14, 42–44]. Furthermore, Aβ peptides are also powerful inhibitors of angiogenesis, in both in vitro and in vivo studies [45]. The capillary network in cerebral cortices has demonstrated severe amyloid plaque accumulation and deposition, compromising the cerebrovasculature with a loss of small cortical arterioles and capillaries [14, 46]. Previous studies have shown that AD patients have increased levels of VEGF in the brain, a potent angiogenic factor necessary for the growth of vascular endothelial cells. This increase of VEGF suggests a compensatory mechanism in response to damaged cerebral structure, even though this mechanism ultimately fails to yield proper angiogenesis [14, 47, 48].

Bone repair following fracture constitutes an interplay between angiogenic and osteogenic pathways. Angiogenesis is necessary for fracture healing to occur and to prevent the onset of osteoporosis. Angiogenesis in actively regenerating calluses supplies the nutrients, oxygen, cytokines, and growth factors necessary for the formation of osteoblasts and osteoclasts, ultimately leading to bone formation [49]. Studies have shown that disruption in angiogenesis precedes the onset of osteoporosis, as inadequate blood flow is linked to impaired bone remodeling and subsequent low bone mass [40]. By a similar mechanism, angiogenesis also precedes osteogenesis. The endothelial cells are arguably the most important components of the vasculature, as they maintain a permeable barrier and allow for the recruitment of hematopoietic cells to the bone site to maintain bone homeostasis and facilitate fracture repair [40, 50–52].

## Bone Mineral Density (BMD)

Lower BMD is associated with an increased risk of developing AD [53]. On the other hand, studies have shown that AD patients have reduced hip BMD and are at twice the risk of developing hip fractures [54, 55]. Large prospective studies have demonstrated an association between reduced BMD and an increased incidence of AD in the elderly [5, 53, 56–58]. Supporting this idea, a recent meta-analysis of three longitudinal studies found that a higher baseline BMD has a significant protective association with incident dementia (new cases of dementia); however, prior bone loss was not found to be associated with incident dementia [59].

A study in a Chinese population examined the potential role of low BMD on the transition from mild cognitive impairment to AD and found a positive relationship between osteoporosis and the decline in cognitive function observed in AD. Subjects in the lowest quartile for BMD were at twice the risk for AD compared to controls. Furthermore, the study revealed that individuals who were identified to have mild cognitive impairment at study onset were more likely to develop AD if they had a low baseline BMD. The study also showed that severe low BMD at baseline was associated with an increased risk of developing AD; this association was seen in both men and women [57]. Together, these studies imply a link between bone density loss and Alzheimer’s that requires exploration of common risk factors to identify a potential root cause.

## The Protective Effect of Estrogen

Dementias have a variety of risk factors, including sex. Depending on the type of dementia, the epidemiology of male-to-female prevalence varies. AD is the most common form of dementia, with females constituting roughly 2/3 of all affected individuals [60]. The increased prevalence of AD in females as compared to men is likely due to the loss of the protective effect of estrogen in females with menopause. Indeed, in the normal brain, estrogen works in the nucleus basalis of Meynert to maintain normal cognitive function [61]. Furthermore, a cross-sectional study conducted in the Netherlands reported that women in the highest quintile of estradiol or estrone were 40% less likely to experience cognitive impairment compared to those in the lowest quintile [62], suggesting a protective effect of estrogen. Studies indicate that estrogen deprivation plays a vital role in the onset of cognitive decline and increased risk for AD in both men and women [61].

A study by Hoskin et al. found that levels of sex hormone–binding globulin (SHBG) were 20% higher in AD patients and that levels of estradiol were significantly reduced, compared to controls [63]. An estimated 37% of estradiol in elderly women circulates in the body bound to SHBG, the form that is postulated to be unable to cross the blood–brain barrier and thus cannot exert effects on the CNS. In other words, roughly 37% of elderly women’s estradiol is unable to be used for the protective effect of estrogen on the brain. Similarly, several observational studies have found an association between increased levels of SHBG and AD [61, 64, 65].

While these studies are promising for elucidating the negative effects of low estrogen levels on increased risk of developing AD in elderly women, there are conflicting studies that suggest a lack of association. The Rancho Bernardo study did not find a significant effect on cognitive test outcome due to bioavailable estradiol [66], and the Rotterdam study found that women with greater bioavailable estradiol levels demonstrated significantly poorer cognitive function [67]. However, these conflicting results may be due to variations in hormone measurement procedures [61].

Estrogen receptors are heavily expressed in osteoblasts, osteoclasts, and osteocytes, making their interaction with estrogen an important factor in the success of bone remodeling throughout the lifetime. Osteoporosis in post-menopausal women is directly related to estrogen deficiency. A deficiency in estrogen leads to increased bone resorption and a negative balance between bone resorption and formation [68]. Estrogen binds to estrogen receptors to inhibit osteoclast formation via the expression of osteoprotegerin. Estrogen can also activate the Wnt/β-catenin signaling pathway to increase osteogenesis. Thus, a lack of estrogen will alter the expression of target genes such as interleukin-1 (IL-1), IL-6, tumor necrosis factor alpha (TNFα), insulin-like growth factor (IGF), and transforming growth factor beta (TGFβ), decreasing osteogenesis. In females, the primary treatment for estrogen deficiency–related osteoporosis is estrogen supplements [68], primarily in the form of transdermal estradiol [69].

When estrogen binds to its receptors, it can also regulate the expression of gene-encoding proteins such as IL-1, IGF, and TGFβ [70]. Estrogen works to upregulate bone morphogenetic protein (BMP) signaling, which promotes mesenchymal stem cell differentiation from pre-osteoblasts to osteoblasts, enhancing bone formation in the remodeling process [68]. Estrogen also suppresses the action of receptor activator of nuclear factor kβ ligand (RANKL) to inhibit osteoclast activity. RANK is expressed on osteoclast precursors, and binding by RANKL promotes osteoclast formation and subsequent resorption. When estrogen binds to osteoclast-expressed estrogen receptors, RANK activity is suppressed [71]. Furthermore, estrogen inhibits the differentiation of osteoclasts and promotes osteoclast apoptosis through the increase of TGFβ production. Thus, estrogen serves to regulate the bone resorption rate.

Osteocytes are the foundational bone material and serve to control bone remodeling and mineralization [68]. The decline in estrogen levels in menopausal women has been associated with bone loss [72, 73] characterized by an increase in both osteoblasts and osteoclasts [74]. In men, low androgen levels result in bone loss and increased bone remodeling [75, 76], in part due to lower levels of estrogen [77]. A study found that in the absence of estrogen receptors, osteocytes were not able to provide an adequate response to received mechanical strain, thus representing a deficiency of osteocyte mechanosensory ability in the absence of estrogen [78].

Estrogen deficiency has major contributions to the pathophysiology of both AD and osteoporosis, affecting both the risk and progression of both diseases. While there has been some conflicting evidence about the role of estrogen in AD, it is reasonable to identify the deficiency of this hormone as a common risk factor between the two conditions, and estrogen may play a role in a shared disease mechanism. Identifying and unraveling the complex relationship between sex hormones and AD progression as well as the shared commonalities of pathways in bone disorders may help in developing potential therapies to improve bone mass while slowing the progression of AD, especially in post-menopausal women.

## Benefits of FSH Blockade

Follicle-stimulating hormone (FSH) is an important regulator in the reproductive systems of men and women, and its blockade is shown to have beneficial effects on inhibiting the hallmarks of AD such as Aβ deposition and p-tau [79••]. Previous studies in mice have shown that FSH works to increase bone mass and enhance thermogenesis, two factors which are dysregulated in AD [80–82]. A study by Xiong et al. demonstrates that FSH accelerates Aβ and tau deposition in the hippocampus and cortical neurons, thus impairing cognition in 3xTg-AD mice. The study shows that blocking the action of FSH in 3xTg-AD mice inhibits the formation of plaque and neurofibrillary tangles, thus alleviating these adverse cognitive symptoms [79••]. Furthermore, recent results indicate that anti-FSH antibody is useful in increasing the bone formation of the femur and spine in mice [83•].

## Neuroinflammation

Current evidence suggests that the progression and severity of AD can be attributed to the immunological mechanisms that occur in the brain [84]. For example, expression of immune receptors, such as triggering receptor expressed on myeloid cells 2 (TREM2) [85] and CD33 [86, 87], has been found to be associated with AD, suggesting that neuroinflammation contributes to the onset and progression of AD [84]. TREM2 is expressed in the microglia of the brain, and the variant R47H has been found to present a significantly higher risk of late-onset AD development [88]. Furthermore, the TREM2 variant Y38C in the brain disrupts the normal functionality of TREM2, causing changes in the microglia morphology and impairing the synaptic plasticity in the hippocampus. The downstream effects of the dysfunction of TREM2 provide an explanation of the events leading to AD and dementia [88]. These downstream effects are discussed in detail by Lee-Gosselin et al., who found in the brains of TREM2^−/−^ mice injected with human tau extract that there was a significant decrease in microglial density compared to controls, as well as diminished tau pathology. This suggests that the experimental mice may not demonstrate a sufficient activated inflammatory response in the presence of tau pathologies, such as aggregation [89]. The observations from Lee-Gosselin et al. suggest that deletion of TREM2 may be beneficial in improving certain hallmarks of AD.

Additionally, it is hypothesized that the formation of neurofibrillary tangles is due to the neurotoxicity seen in neuroinflammation [16]. Furthermore, activated microglia and astrocytes seen in the inflammatory process surround the amyloid plaque depositions, resulting in higher levels of inflammatory mediators than observed in non-AD brains [90]. Reactive astrogliosis has been shown to occur in many neurodegenerative tauopathies, such as AD [91]. Taken together, these observations implicate neuroinflammation and the glial response as contributors to the damage of neurons and ultimately AD [92, 93].

Limited work has been conducted looking at the link between neuroinflammation and bone. TREM2 is expressed on osteoclasts, regulating the rate of osteoclastogenesis, and a study by Otero et al. reports that TREM2^−/−^ mice exhibit osteopenic phenotype resembling the Nasu-Hakola disease [94, 95]. Furthermore, the TREM2 R47H variant has been implicated in low bone mass and skeletal muscle strength seen in TREM2^R47H/+^ mutant female mice, independent of central nervous system pathology [96].

## Oxidative Stress

Reactive oxygen species (ROS) are free radicals that regulate cellular homeostasis and can be formed from both endogenous and exogenous sources. Endogenous sources of ROS include the mitochondrial respiratory chain and various enzymatic reactions, while exogenous sources are various stressors such as ionizing radiation and oxidizing chemicals [97]. Normally, ROS are important messengers in cell signaling, but at high concentrations, they can cause damage to cells leading to necrosis and apoptosis [97].

Oxidative stress is a major contributor to the progression of AD [97], with ROS being a critical player in the pathology of AD [98]. Oxidative stress has been shown to expedite aging and accelerate the onset of AD. The progressive cell loss due to oxidative stress can lead to the onset of neurodegenerative diseases; in AD, this causes abnormal aggregation of amyloid proteins [97]. In patients with AD, there is significant oxidative damage to brain tissue [99, 100], which leads to the upregulation of Aβ and p-tau formation [97, 99]. Double bond peroxidation in polyunsaturated neuronal lipid products forms molecules that stimulate p-tau [98, 101–105].

Oxidative stress has also been implicated as a causative factor in the diminished BMD in osteoporosis [106]. Kimball et al. cite four avenues through which oxidative stress affects the pathway of bone metabolism: (1) upregulation of osteoclastogenesis, (2) decreased osteoprogenitor differentiation, (3) decreased osteoblast activity, and (4) increased osteoblast and osteocyte apoptosis [106]. Oxidative stress causes increased osteoclastogenesis through the upregulation of RANKL and downregulation of osteoprotegerin; these two factors are an osteoclast activator and inhibitor, respectively [107–109], and occur via the Wnt/β-catenin pathway [107]. A study has shown that hydrogen peroxide–induced oxidative stress decreases osteoblast differentiation, thus inhibiting the formation of new bone [110]. Decreased osteoblast differentiation leads to decreased osteoblast activity and thus decreased osteoprotegrin production [106], ultimately ceasing regulation of osteoclast activity. Osteoblast and osteocyte apoptosis increase with oxidative stress, further inhibiting osteogenesis [106], while stimulating osteoclastogenesis via decreased osteoblastic cytokine activity [33, 111–115].

## Therapies

There are currently three classes of FDA-approved drugs to treat AD: cholinesterase inhibitors, NMDA antagonists [8], and monoclonal antibodies. Acetylcholinesterase inhibitors function to block the breakdown of acetylcholine, thereby increasing the levels of acetylcholine in the synaptic cleft [116–118]. This medication helps to reduce the effects of the reduced cholinergic transmission throughout the brain due to the destruction of acetylcholine-producing cells in AD [8]. NMDA antagonists work to prevent cell death and synaptic dysfunction caused by excitotoxic overactivation of the NMDA receptor and subsequent increased levels of calcium [119, 120]. These two drugs are effective in managing the symptoms of AD but do not cure the disease [8, 17]. Due to the lack of disease-modifying therapies, research has focused on prevention or risk reduction of AD [121]. Studies have shown that lifestyle modifications such as physical activity, diet, and cognitive training can increase or maintain cognitive function and reduce new cases of AD in the elderly [8, 122]. Monoclonal antibodies have shown some promise in slowing the progression of AD. Trials using the monoclonal antibody aducanumab reported that high doses of the drug had the potential to slow the cognitive decline seen in AD, and the drug was given conditional FDA approval in 2022 [123]. Lecanemab, a humanized IgG1 monoclonal antibody [18•] which received FDA approval in July 2023, was found to reduce the markers of amyloid plaques in early-onset AD and led to less cognitive decline after 18 months of use, when compared to placebo. While this is the first drug that demonstrates slowing of AD progression to receive full FDA approval, ongoing studies are being conducted to determine the overall safety of the drug [18•].

In contrast, lifestyle modifications are the first-line treatment for the prevention or treatment of osteoporosis [124]. These modifications include eating a healthy and varied diet with calcium-rich and vitamin-rich foods [125], as well as reducing alcohol consumption and avoiding smoking [126]. However, these lifestyle modifications may not be enough for some patients, and thus, there are a variety of pharmaceutical options. There are two main treatment categories: anabolic treatments, which activate osteoblasts [127], and inhibitors of catabolism, which inhibit osteoclast-mediated resorption [128]. The most commonly prescribed anabolic treatments are parathyroid hormone (PTH) derivatives [124], although few patients receive full-length PTH and administration of PTH derivatives is given intermittently. PTH is a hormone known to promote bone resorption when administered continuously and promote bone regeneration when administered intermittently, regulating endochondral bone development [124], while maintaining higher BMD [129]. The most prescribed anti-catabolic treatments for osteoporosis are bisphosphonates. Bisphosphonates inhibit osteoclast activity and induce osteoclast apoptosis, thereby blocking bone resorption and stopping bone loss [124]. Thus, these two treatment categories serve to target osteoporotic pathologies at the mechanistic level to slow progression.

## Increased Risk of Fractures Following Alzheimer’s Disease Diagnosis

It is well known that fracture is the most common sequela of osteoporosis, but it has been found to be a complication in AD as well. Research has shown that individuals with AD are more than twice as likely to sustain fractures at disease onset, despite having comparable risk to controls prior to the onset of AD symptoms [130•]. This increased risk occurs as soon as the first year of disease onset [131, 132]. The main risk factors for the increased incidence of hip fractures in AD patients are low BMD [4, 133], low concentrations of serum ionized calcium, and low concentrations of 25-hydroxyvitamin with compensatory hyperparathyroidism [134, 135]. Following a hip fracture, functional recovery is poor in AD patients [134, 136, 137], with individuals having a significantly lower ambulatory level [138] and greater risk of immobilization [139] compared to controls. Furthermore, AD patients have a higher risk of post-fracture mortality [140]. Another study found that individuals with dementia were at an even higher risk of developing a hip fracture if they also had diagnosed osteoporosis [54]. This idea is supported by the fact that individuals with AD have an increased risk of falling and subsequent fracture, with co-occurring osteoporosis being one of the strongest predictors of hip fractures [4, 54, 141, 142].

## Increased Risk of Developing Dementia/AD Following Fracture Incidence

A 2020 observational study found that an incidence of distal radius, hip, and spine fractures increased the risk of developing dementia in individuals greater than 60 years of age [143]. A retrospective study found that, after a 12-year follow-up period, the overall incidence rate of dementia following fracture was 41% higher than in individuals who did not experience a fracture incidence [3]. Interestingly, the degree of increased risk varies depending on the fracture site, with hip fractures being the greatest at 60% higher risk of developing dementia. Comparatively, those with vertebral fractures had a 47% higher risk, those with thigh/leg/ankle fractures had a 35% higher risk, and those with an upper limb fracture exhibited a 29% increased risk [3].

There are several factors, both during the fracture incidence and healing process, that have been proposed to predispose or increase the risk of one developing dementia. It is hypothesized that fractures can predispose individuals to developing dementia due to the inflammatory process and reactive oxidative stress associated with fracture healing [143], as well as impaired balance [144–146] and vestibular asymmetry [147–149]. Following a fracture incidence, the inflammatory cytokines TNFα and IL-6 are elevated [150] in both the cerebrospinal fluid and peripheral blood [151, 152], and these two factors have been implicated in dementia [153]. Furthermore, ROS levels increase during fracture healing [154], which may lead to oxidative brain injury, thus increasing the risk of dementia [155].

Following a fracture, the complications of recovery may increase the risk of dementia via decreased physical activity and postoperative delirium [143]. Observed functional mobility declined in patients following a fracture [156], and a retrospective study found that roughly 32% of hip fracture patients who experienced postoperative delirium were later diagnosed with dementia [157].

Even though there are some observational clinical studies showing an association between the incidence of fractures and an increased risk of AD, to date, no studies exist in either humans or animal models of AD showing a link between fractures and AD progression. Due to a lack of these studies, it is imperative that this less understood link be explored further to uncover the intertwined pathways between fractures, bone health, inflammation, and AD.

## Conclusion

**Fig. 1:**
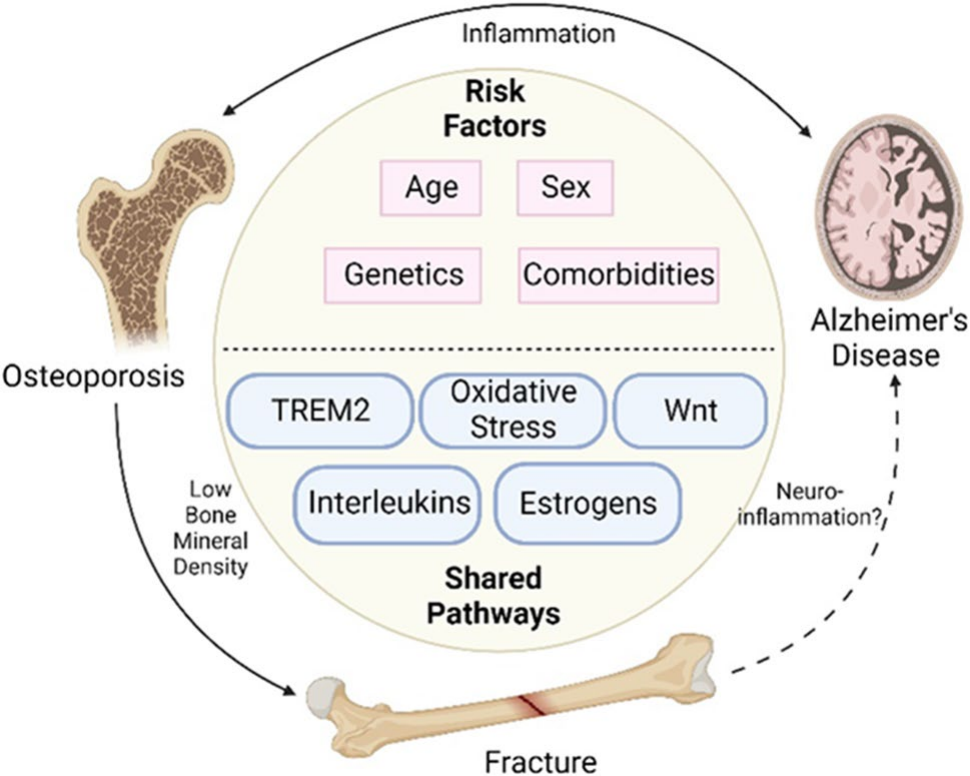
Alzheimer’s disease and osteoporosis share many commonalities in their disease processes, such as inflammation, oxidative stress, estrogen deficiency, and, therefore, potential for shared therapeutics

As illustrated in Fig. 1, Alzheimer’s disease and osteoporosis share many of the same disease mechanisms, such as altered angiogenesis, low BMD, a decrease in estrogen levels, neuroinflammation, and increased oxidative stress. The diseases both involve some of the same signaling pathways, and some of the characteristic molecular hallmarks of AD, such as Aβ and APP, have been shown to play a role in osteoporosis as well. One disease often predisposes an individual to the other, and many elderly patients concurrently have both AD and osteoporosis. The co-occurrence of these two degenerative diseases provides a great negative impact on the individual, namely, an increase in fractures and subsequent decreased mobility. Fractures have been shown to occur at greater rates in AD and osteoporosis patients compared to controls, largely due to the low BMD seen in both diseases. Interestingly, recent evidence suggests that an incidence of fracture also predisposes one to developing dementia, suggesting further commonalities between these two common geriatric diseases. Further exploration on fracture incidence causing AD onset is warranted, as this could uncover additional mechanistic commonalities and provide more insight into the pathogenesis of AD. Osteoporosis, AD, and fracture are debilitating ailments with recently uncovered similarities in pathophysiology. It is important to note that mouse models exploring such links have a variety of limitations, largely due to incomplete replication of the remodeling patterns seen in humans. As a result, there is a need for more human studies. That said, a limitation of human studies is the difficulty of drawing inferences related to pathways and mechanisms involved. Thus, both preclinical and clinical studies are essential to tackle these debilitating diseases. Finally, understanding the complex relationship between osteoporosis, AD, and fracture healing will be crucial to the development of therapies to improve the lives of people everywhere, especially the elderly.
